# Does centrifugation matter? Centrifugal force and spinning time alter the plasma metabolome

**DOI:** 10.1007/s11306-016-1109-3

**Published:** 2016-09-15

**Authors:** Dorothea Lesche, Roland Geyer, Daniel Lienhard, Christos T. Nakas, Gaëlle Diserens, Peter Vermathen, Alexander B. Leichtle

**Affiliations:** 1 University Institute of Clinical Chemistry, Inselspital, Bern University Hospital, University of Bern, INO F, 3010 Bern, Switzerland; 2 Graduate School for Cellular and Biomedical Sciences, University of Bern, Bern, Switzerland; 3 Laboratory of Biometry, University of Thessaly, Volos, Greece; 4 Departments of Clinical Research and Radiology (AMSM), Inselspital, Bern University Hospital, University of Bern, Bern, Switzerland

**Keywords:** Centrifugation, Plasma, Metabolome, Relative centrifugal force, Spinning time, Preanalytics

## Abstract

**Background:**

Centrifugation is an indispensable procedure for plasma sample preparation, but applied conditions can vary between labs.

**Aim:**

Determine whether routinely used plasma centrifugation protocols (1500×*g* 10 min; 3000×*g* 5 min) influence non-targeted metabolomic analyses.

**Methods:**

Nuclear magnetic resonance spectroscopy (NMR) and High Resolution Mass Spectrometry (HRMS) data were evaluated with sparse partial least squares discriminant analyses and compared with cell count measurements.

**Results:**

Besides significant differences in platelet count, we identified substantial alterations in NMR and HRMS data related to the different centrifugation protocols.

**Conclusion:**

Already minor differences in plasma centrifugation can significantly influence metabolomic patterns and potentially bias metabolomics studies.

**Electronic supplementary material:**

The online version of this article (doi:10.1007/s11306-016-1109-3) contains supplementary material, which is available to authorized users.

## Introduction

With current technologies, thousands of small molecules can be obtained from a single biological sample at the same time (Psychogios et al. 2011; Wishart et al. 2013). Since obtaining blood samples is minimally invasive and sampling devices are widely available in medical offices and at hospitals, blood plasma and serum are the most commonly used sample matrices in human metabolomic studies (Vuckovic 2012; Guder and Narayanan 2015). Among other important pre-analytical variables, a potential source of variation is the preparation of plasma from whole blood by centrifugation. Protocols are usually highly standardized within labs, whereas the relative centrifugation force (RCF) and spinning time can substantially vary between labs and projects (Suchsland et al. 2014). The World Health Organization recommends a protocol applying 2000–3000×*g* for at least 15 min to prepare cell-free plasma (World Health Organization 2002) and it is claimed that centrifugation force and spinning time could be reciprocally adjusted (Thomas 2012). Regarding subsequent non-targeted metabolomic studies, varying centrifugation conditions may generate considerable bias. As previously reported, differences in pre-analytical conditions such as sample collection and storage introduce substantial bias (Leichtle et al. 2013). Different centrifugation protocols influence the amount of platelets remaining in the plasma, potentially altering the biochemical signature of the samples (Daves et al. 2014). Hence, we evaluated the difference of the metabolic profile of human plasma samples introduced by two different standard centrifugation protocols routinely used in our coagulation and clinical chemistry core lab. To address this question, we applied a comprehensive approach using non-targeted nuclear magnetic resonance (NMR) spectroscopy and ultra-high performance liquid chromatography with quadrupole-time-of-flight mass spectrometry (UPLC-QTOF) techniques. Comparison of the cell count before and after centrifugation provided insight on the influence of remaining cells to the sample composition.

## Materials and methods

### Sample collection and centrifugation

Two EDTA blood samples (S-Monovette^®^ 2.7 ml, K3 EDTA; Sarstedt, Nümbrecht, Germany) were collected from each of ten apparently healthy volunteers using Safety-Multifly^®^-needles (Sarstedt, Nümbrecht Germany). Immediately after blood sampling, automated blood cell count was performed on a Sysmex XN9000 instrument (Sysmex Suisse, Horgen, Switzerland). Both blood collection tubes of each patient were centrifuged using two different protocols, either at 1500×*g* for 10 min or at 3000×*g* for 5 min at 20 °C, respectively. Automated blood cell count was repeated after centrifugation in the plasma supernatant. Two plasma aliquots of each centrifugation condition were instantly frozen at −80 °C in FluidX external screw cap 525 µl cryovials (FluidX, Wehrheim, Germany) until sample preparation and analysis.

### NMR analyses

Plasma aliquots (250 µl) were mixed with 300 µl PBS (pH 7.4) and transferred into standard 5 mm NMR tubes. ^1^H-NMR experiments were performed on the 20 plasma aliquots in randomized order for metabolic profiling. A Bruker Avance II spectrometer (Bruker, Karlsruhe, Germany) operating at a resonance frequency of 500 MHz for ^1^H and equipped with a 5 mm ATM BBFO probe with z-gradient was used to acquire the spectra employing a PROJECT (Periodic Refocusing of J-Evolution by Coherence Transfer) sequence (Aguilar et al. 2012). Each spectrum was acquired at 300 K applying 64 transients, a spectral width of 10 kHz, a data size of 64 K points, an acquisition time of 3.28 s, and a relaxation delay of 5 s. The spectra were processed using the Bruker Topspin software (version 3.1, patch level 6). Processing included exponential weighting with a line broadening factor of 0.5 Hz, Fourier-transform, and manually phasing. A home-written Matlab program (using MATLAB R2012a, Mathworks^®^) was used for bucketing and Probabilistic Quotient Normalization (PQN) of the data.

### UPLC-QTOF analyses

Plasma aliquots were prepared by protein precipitation using pre-chilled (−20 °C) methanol/acetonitrile 1:1 (v/v) and analyzed in randomized injection order on a QTOF MS (Synapt G2-Si HDMS, Waters Corp., Milford, USA) coupled with preceding chromatographic separation on an UPLC Acquity system (Waters) using an Acquity UPLC HSS T3 C18 column. The mobile phases comprised of 0.1 % formic acid in LC–MS-grade water as well as methanol containing 0.1 % formic acid. A linear gradient over 14 min changing from polar to organic phase was applied (for details see Supplementary Information 1).

### Statistical analysis

Statistical analysis was performed using the “R” software (version 3.2.3; https://www.R-project.org/) and SIMCA (version 14, MKS Instruments AB, Malmo, Sweden). Normality testing for cell count parameters was carried out using the Anderson–Darling test. To compare the hemograms before and after centrifugation the paired Wilcoxon signed rank test with continuity correction was performed. Multilevel sparse partial least squares discriminant analyses (sPLS-DA) was used on NMR bucket data (Gonzalez et al. 2011). NMR buckets were assigned to metabolites based on literature (Liu et al. 1996) as well as on additionally performed 2D NMR measurements (data not shown). Data generated on the UPLC-MS systems (i.e., raw-files) were directly imported into Progenesis QI (Nonlinear Dynamics, Newcastle upon Tyne, UK) for peak picking and run alignment. In addition, raw data was converted into mzXML-format using Proteowizard (Chambers et al. 2012) for processing with the ‚xcms’-package for R (https://masspec.scripps.edu/xcms/download.php) according to previously published protocols (Patti et al. 2012; Tautenhahn et al. 2008; Smith et al. 2006; Benton et al. 2010). After extraction of the peak table, an sPLS-DA was performed on the xcms peak data as well. Metabolic features from UPLC-QTOF MS analysis were tentatively assigned using the METLIN database with Δm/z of 0.3 Da (Smith et al. 2005). PCA was performed using the ‘ropls’ package for R.

## Results and discussion

Before centrifugation, samples in both protocol groups did not differ in their apparently normal hemogram results (cf. Supplementary Information, Table 1). After centrifugation, plasma samples originating from both protocols differed in thrombocyte count (p = 0.05, median_1500×*g*; 10min._ = 137.5 G l^−1^ (IQR 83.0–162.1), median_3000×*g*; 5min._ = 59.5 G l^−1^ (IQR 12.6-81.2), cf. Supplementary Information, Fig. 2). Already a simple hemogram revealed significant differences between centrifugation protocols regarding the residual thrombocyte count, suggesting consequent alterations in the metabolome. According to the literature, both protocols should yield the same results (centrifugal force and spinning time reciprocally related by factor 2, Thomas 2012). As 3000×*g*/5′ resulted in lower platelet counts, we assume that the centrifugal effect of g-force is overcompensating the effect of the spinning time.

**Fig. 1:**
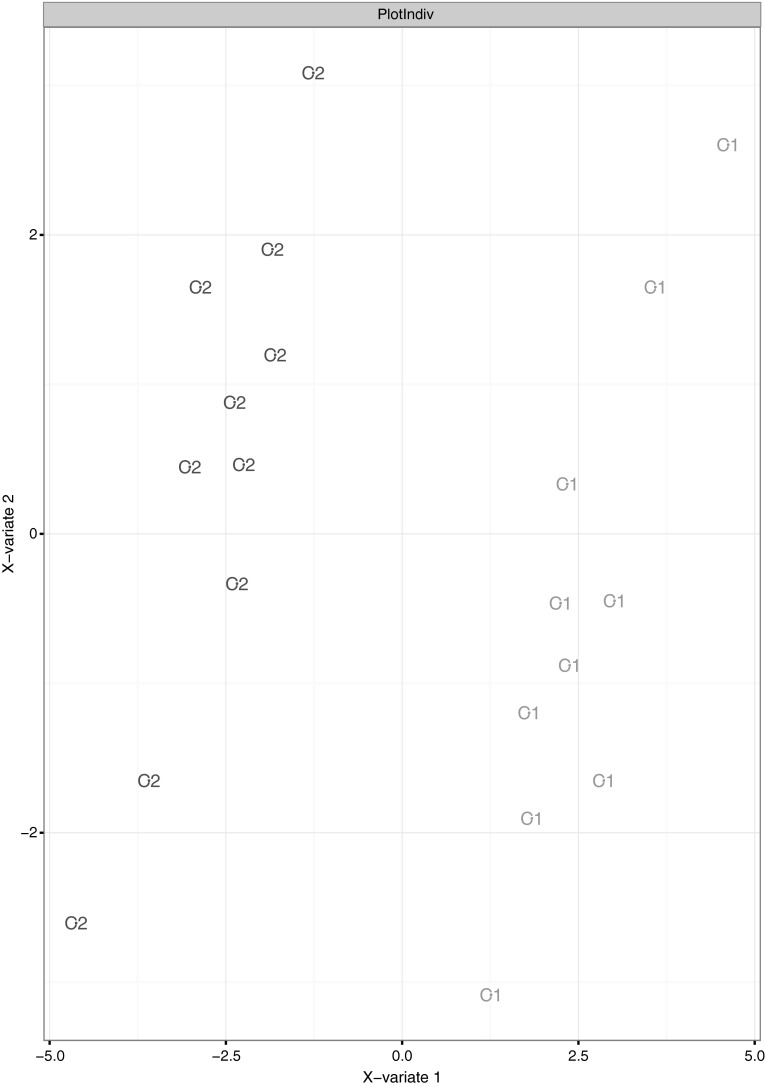
sPLS-DA plot of the individuals of both centrifugation groups (C1 1500×*g*, 10 min; C2 3000×*g*, 5 min) for the NMR data

In NMR analysis we could extract a total of 186 spectral buckets. Principal component analysis (PCA) on these buckets demonstrated high metabolic reproducibility of plasma aliquots, i.e. clustering of scores from plasma of the same subjects (cf. Supplementary Information, Fig. 1a). However, a subsequent multilevel sPLS discriminant analysis yielded complete separation of both post-centrifugation groups (Fig. 1). Responsible for this separation was mainly glutamine, present in 5 of the top 20 buckets (4 × lower in group C1 than in group C2, 1 × higher in group C1 than in group C2, Metabolomics Standards Initiative (MSI) level 2 identification), which is actively metabolized in human platelets as a preferential mitochondrial oxidative substrate (Vasta et al. 1995). We assume that concentration in the sample extract might be higher in case of more remaining thrombocytes after centrifugation.

**Fig. 2:**
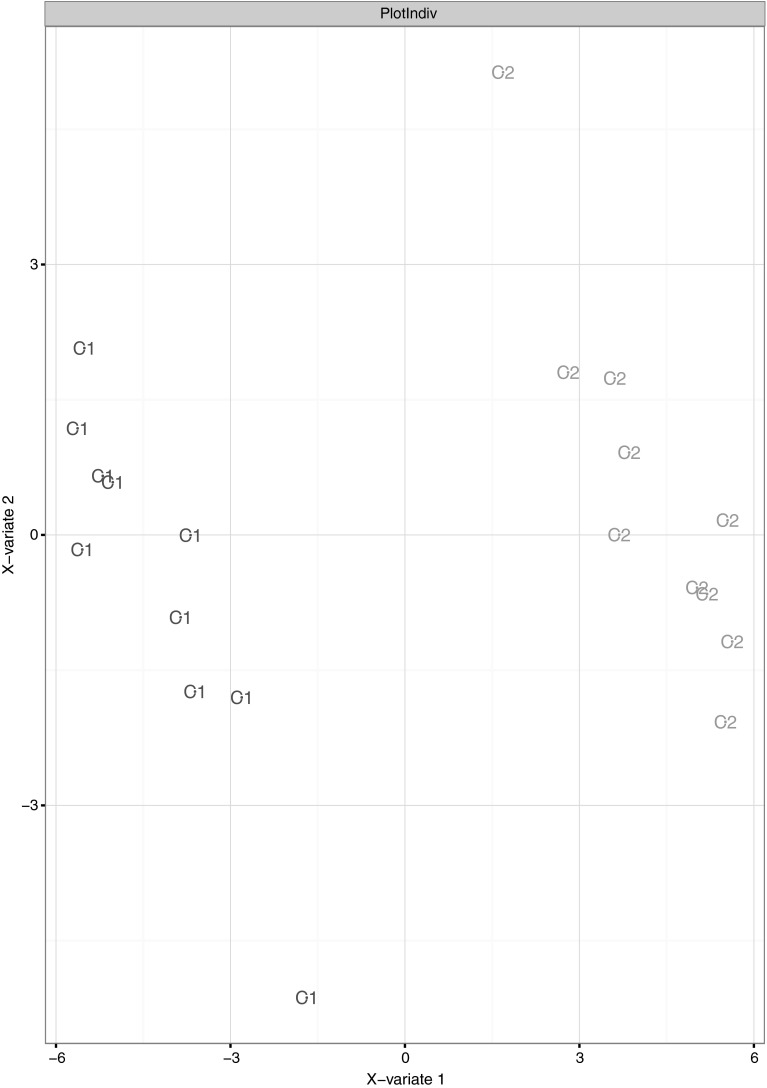
sPLS-DA plot of the individuals of both centrifugation groups (C1 1500×*g*, 10 min; C2 3000×*g*, 5 min) for the UPLC-QTOF data

We recorded 20 post-centrifugation spectra using UPLC-QTOF MS in positive as well as negative ionization mode, 10 for each centrifugation protocol. The positive mode spectra were converted to mzXML files to be analyzed with the ‚xcms’-package for R. After peak alignment, retention time correction, and peak filling, we extracted a peak list and performed a multilevel sPLS-DA (Fig. 2) on the transposed table from xcms. PCA did not reveal separation of the centrifugation groups (Supplementary Information, Fig. 1b). From the metabolic features of xcms data analysis responsible for group separation (n = 43, p < 0.2), about two-third could be matched to features extracted by Progenesis QI (retention time and high resolution MS data matched; data not shown). Search in METLIN database and putative assignment of identities revealed mainly lipid species. Responsible for the sPLS-DA based group separation of the positive mode MS xcms data were, among others, the peaks with m/z 161.0586 at RT 0.80 min, m/z 787.6515 at RT 12.52 min and m/z 835.6451 at RT 12.63 min. The latter two compounds appear to be a sphingomyelin SM(40:1) and the sodium adduct of a sphingomyelin SM(42:2), respectively, according to the mass and fragmentation spectra from high energy scan in positive and negative ionization mode (MSI level 3 identification). For both, NMR and UPLC Q-TOF analyses, PCA did not show clustering for age or sex (data not shown), separating buckets and peaks are displayed as Supplementary Information, Fig. 3.

Since there is currently no consistent recommendation regarding centrifugation conditions in metabolomics studies, selection criteria for spinning conditions represent a balancing between gentle cell handling and shorter turnaround time. It is well known that centrifugation conditions vary between laboratories, but despite the potential influence on many subsequent analyses, centrifugation conditions are still a neglected “blind spot”in otherwise rigid pre-analytical standardization (Guder and Narayanan 2015; Brauer et al. 2010; Vuckovic 2012; Yin et al. 2015). In our laboratory, both centrifugation settings are applied in clinical routine diagnostics, depending on the requested analyses (e.g., lower RCF for coagulation samples). We were able to identify significantly different buckets and peaks that provide discriminatory power between these two centrifugation protocols in multilevel sPLS-DA using NMR as well as UPLC-QTOF MS measurements, respectively. Moreover, in the NMR analyses free glutamine content seems to be associated with centrifugation conditions. Using non-targeted UPLC-QTOF MS analyses we primarily identified different lipid classes (i.e., glycerophosphocholines and sphingomyelins) to be affected by selection of a certain centrifugation protocol. Glutamine is known to show a high plasma variability due to the active hydrolysis of glutamine in platelets (Guder et al. 2009), whereas lipids are affected by the residual cell counts at lower and increased cell disruption at higher centrifugation force. Due to the paired study design with two identical primary samples, a timely standardized pre-analytical handling of each sample, and the randomized batch-wise analytical procedures, we may largely exclude additional sources of bias that might have interfered with the sample composition. While the fold changes in single metabolites identified with both strategies are generally low, their combined effect leads to perfect separation of both groups. In case of a small biological difference among analysis groups and use of different centrifugation protocols, these *per se* minor changes might substantially contribute to group separation and lead to biased interpretation.

## Conclusion

Our study demonstrates that different routinely used centrifugation conditions exert significant effects on the results of non-targeted metabolomics analyses using NMR and UPLC-QTOF MS. Therefore it is necessary to carefully standardize the centrifugation protocols to ensure comparability of samples, especially when it comes to multi-center studies or long-term sample storage in a biobank. If centrifugation conditions, for example across study centers, are not properly reviewed, study results might be considerably biased and irreproducible. Detailed centrifugation protocols should be a regular part of any publication covering plasma sample preparation, especially in the field of metabolomics.

## Electronic supplementary material

Below is the link to the electronic supplementary material.

Supplementary material 1 (DOCX 306 kb)
