# Supplementary material for: Does centrifugation matter? Centrifugal force and spinning time alter the plasma metabolome
Source: Metabolomics. 2016 Sep 15;12(10):159. doi: 10.1007/s11306-016-1109-3 (PMC5025507; doi:10.1007/s11306-016-1109-3)
Supplement: Supplementary file 1 — Supplementary material 1 (DOCX 306 kb) [file 11306_2016_1109_MOESM1_ESM.docx]

Supplementary Information to

**Short communication: Does centrifugation matter? Centrifugal force and spinning time alter the plasma metabolome.**

**UPLC-qTOF analyses**

Plasma aliquots were thawed on ice during 60 min, vigorously vortexed (15s) and spun down for 30 s at 500×g. Sample extraction was performed by adding 280 μl of pre-chilled (-20°C) methanol/acetonitrile 1:1 (v/v) to 20 μl of each plasma samples in a 500 μl 96-well plate (Eppendorf, Hamburg, Germany). Plate was gently shaken for 90 s and subsequently stored at -80°C to enhance protein precipitation. The extraction plate was centrifuged for 20 min at 4°C and 4400×g, followed by transfer of 150 μl supernatant to a new 500 μl 96-well plate and rerun of centrifugation at same conditions. The supernatant (100 μl) was transferred into a new 96-well plate, the plate heat sealed with a pierceable foil (ClearASeal Pierce, Chameleon XT, KBiosystems). Quality control (QC) samples were generated by pooling 15 μl of each extracted sample, vigorously mixing and transfer to LC glass vials. All samples including QC samples were stored at -20°C until non-targeted metabolomics profiling analysis. Samples were analyzed in randomized injection order on a quadrupole-time-of-flight (QTOF) mass spectrometer (Synapt G2-Si HDMS, Waters Corp., Milford, USA) coupled to an ultra-performance liquid chromatography (UPLC) Acquity system (Waters) using reverse-phase Acquity UPLC HSS T3 C18 column, 100Å, 1.8 µm, 1 mm X 100 mm (Waters). For UPLC separation, the mobile phase comprised of (A) LC-MS-grade water with 0.1% formic acid as well as (B) methanol containing 0.1% formic acid. Autosampler temperature was set to 6°C and column temperature to 50°C. Each sample was resolved with a linear gradient elution for 15 min at a flow rate of 0.17 ml min^-1^ as follows: 100% mobile phase A for 1 min, changing to 99% B within 10 min, holding at 99% B for 2 min, and finally returning to 100% A and equilibrating for 1.9min. The eluent was introduced into the mass spectrometer Waters Q-TOF Synapt G2-S, operating in either negative ion (ESI -) or positive ion (ESI+) electrospray ionization modes, scanning between m/z 50 and 1200 amu. Standard mix sample was injected (n=4) at the beginning and at the end of analysis run to control for system stability and mass accuracy (i.e., deviation in retention times, Δ m/z < 1.5 mDa). System equilibration was performed by replicate injection of QC samples (n=10). Batch effects and instrument drift were monitored using QC sample injection after every 4 plasma sample injection (n=7) together with the last QCs from system equilibration (n=3). To maintain mass accuracy, Leucin-Enkephalin ([M-H] - 554.2620 and [M+H] - 556.2766) at a concentration of 0.2 µg mL dissolved in 50% acetonitrile was used as for lock mass correction (infusion rate 20 µL min^-1^). Instrument control and sample management was done via MassLynx (version 4.1, Waters).

|  | Descriptive Statistics |  |
| --- | --- | --- |
|  | **Mean (±sd)** | **Min-Max** |
| *Age* | 35.7 (±15.6) | 20-62 |
| *Sex* | Male=4/10 (40%) | (p=0.754 binomial) |
| *N* | 10 |  |
|  |  |  |
|  | **Before Centrifugation** | **1500g; 10’** |
| *Hemoglobin (g/L)* | 139 (±9.428) | 123-155 |
| *Hematocrit (L/L)* | 0.404 (±0.024) | 0.36-0.43 |
| *Erythrocyte count (T/L)* | 4.66 (±0.370) | 3.9-5.05 |
| *MCV (fL)* | 86.8 (±3.011) | 81-92 |
| *MCH(pg)* | 30 (±1.247) | 28-32 |
| *MCHC (g/L)* | 344.6 (±9.652) | 333-360 |
| *RDW (%)* | 12.51 (±0.486) | 11.9-13.3 |
| *Leukocytes (G/L)* | 7.14 (±1.458) | 4.2-9.1 |
| *Thrombocytes (G/L)* | 224.6 (±18.46) | 186-248 |
| *MPV (fL)* | 10.43 (±0.445) | 9.6-11.2 |
|  |  |  |
|  | **Before Centrifugation** | **3000g; 5'** |
| *Hemoglobin (g/L)* | 139.6 (±9.640) | 123-155 |
| *Hematocrit (L/L)* | 0.407 (±0.023) | 0.36-0.43 |
| *Erythrocyte count (T/L)* | 4.680 (±0.367) | 3.9-5.05 |
| *MCV (fL)* | 87 (±3.127) | 81-92 |
| *MCH (pg)* | 30 (±1.247) | 28-32 |
| *MCHC (g/L)* | 343.8 (±9.508) | 335-361 |
| *RDW (%)* | 12.55 (±0.497) | 12-13.4 |
| *Leukocytes (G/L)* | 7.14 (±1.474) | 4.3-9.2 |
| *Thrombocytes (G/L)* | 225.8 (±18.122) | 191-253 |
| *MPV (fL)* | 10.44 (±0.420) | 9.6-11.1 |

Supplementary Table 1: Descriptive baseline statistics of the study cohort (before centrifugation). Abbreviations: MCV mean cellular volume, MCH mean cellular hemoglobin, MCHC mean cellular hemoglobin concentration, RDW relative distribution width, MPV mean platelet volume. No significant differences between centrifugation groups were observed. All participants are caucasians. sd – standard deviation

| **A**  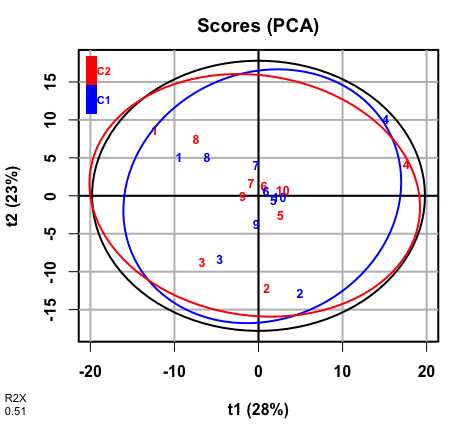 | **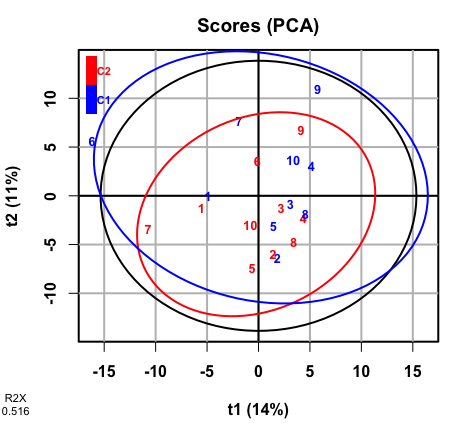B** |
| --- | --- |

Supplementary Figure 1: PCA score plots for both centrifugation protocols (C1: 1500g/10’ blue, C2: 3000g/5’ red) for individuals (numbers), analyzed with NMR (panel A) and UPLC Q-TOF (panel B).

Supplementary Figure 2: Thrombocyte (platelet, „Thc“) counts before and after centrifugation for both centrifugation protocols. We observed no significant difference before but a marginally significant (p=0.05) difference after centrifugation.

| **A**  **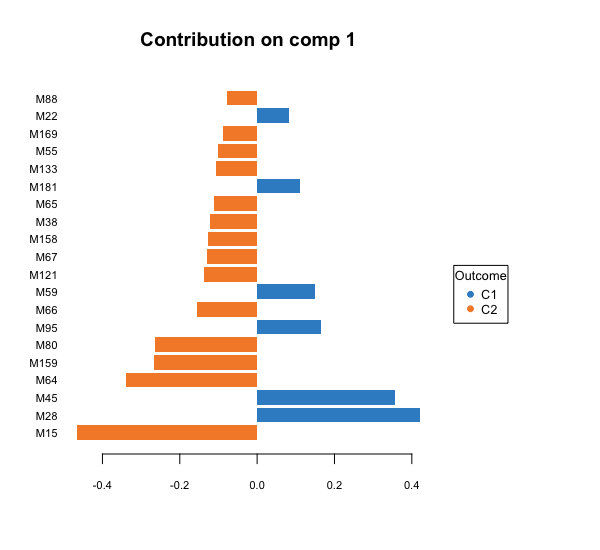** | **B**  **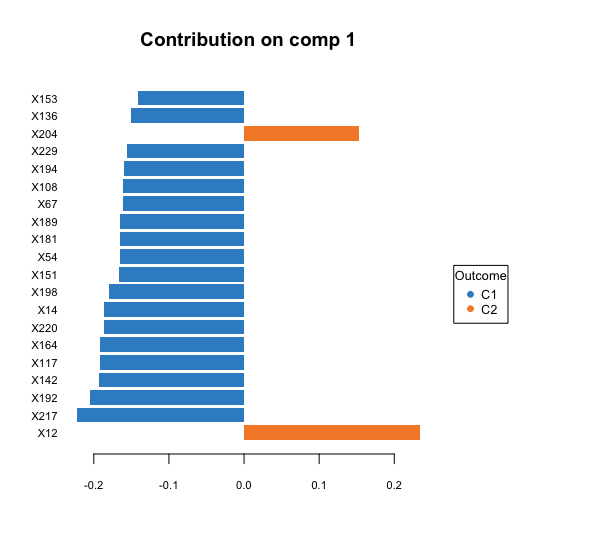** |
| --- | --- |

Supplementary Figure 3: sPLS-DA component contribution plots for component 1 for NMR buckets (panel A, depicted are the top-20 buckets. Buckets M#45, 64, 66, 67, and 65 could be traced back to glutamine) and UPLC Q-TOF Peaks (panel B, X12 corresponds to M161.0_R0.8, X217 to M835.6_R12.6, and X164 to M787.7_R12.5). Outcome refers to the centrifugation protocol (C1: 1500g/10’ blue, C2: 3000g/5’ red).
